# Predictors of health-related quality of life among rural adult residents of western Maharashtra, India

**DOI:** 10.3389/fpubh.2026.1699024

**Published:** 2026-02-02

**Authors:** Jayashree Gothankar, Sanjivani Patil, Medha Bargaje, Rupeshkumar Deshmukh, Ashwini Devane, Prakash Doke

**Affiliations:** 1 Department of Community Medicine, Bharati Vidyapeeth Deemed University Medical College, Pune, India; 2 Central Research and Publication Unit, Bharati Vidyapeeth Deemed University Medical College, Pune, India; 3 Department of Pulmonary Medicine, Bharati Vidyapeeth Deemed University Medical College, Pune, India

**Keywords:** BMI, chronic disease, EQ-5D-5L, health-related quality of life, predictors, rural adults, rural health

## Abstract

**Introduction:**

Health-related quality of life (HRQoL) is a multidimensional measure encompassing physical, psychological, and social functioning. Information on its predictors will help to identify the strategies for improving the quality of life of the affected population. The objective of the study was to determine the health-related quality of life (HRQoL) of rural adults using the EQ-5D-5L index and to identify its key predictors.

**Methods:**

This cross-sectional census enumeration study included all eligible residents from all 35 villages under *Male* Primary Health Centre (PHC) in Mulshi block, Pune district, Western Maharashtra, India. All permanent residents aged 30 years or older who consented to participate were included in the study. The trained Accredited Social Health Activist (ASHA) workers administered a pretested, validated questionnaire during house-to-house visits. They collected sociodemographic, housing information, tobacco use, and morbidity patterns among these residents. Multivariate regression analysis was utilized to determine the predictors of HRQoL.

**Results:**

Of 11,348 participants enrolled (with a response rate of 93.4%), the mean EQ-5D-5L index score was 0.94 ± 0.17 (SD); 36.8% reported some to severe impairments in at least one dimension. The most affected dimensions were pain/discomfort (28.3%) and anxiety/depression (21.9%). Participants over 60 years old, illiterate, non-earning, those with chronic morbidities, and those who use unclean fuel were found to be significantly associated with impairement in EQ-5D-5L. These factors affected nearly all dimensions of the EQ-5D-5L.

**Conclusion:**

The study demonstrates that approximately one-third (36.8%) of the rural adult population experiences impairment in at least one dimension of health-related quality of life. The key factors were age above 60 years, illiteracy, non-earning status, and those with at least one chronic morbidity. These findings have significant implications for targeted public health interventions that can address modifiable risk factors and enhance chronic disease management in rural areas.

## Introduction

Health-related quality of life (HRQoL) is a multidimensional concept used to examine the impact of health status on an individual’s overall quality of life, encompassing both physical and mental aspects of their well-being (1–3). In this context, the rural adults across the world are experiencing multiple chronic conditions increasingly coexisting in the same individual. The most typical morbidities among rural adults are hypertension, diabetes, arthritis, and chronic respiratory diseases, which range from 48% to 72% (4–8).

This disproportionate concentration of disease burden highlights a critical gap in global health equity, underscoring the need for urgent policy attention. Consequently, the World Health Organization (WHO) prioritizes health equity as a crucial tool for sustainable development, with a particular emphasis on reducing disparities in rural and resource-limited settings (9). Understanding health-related quality of life (HRQoL) in these populations is therefore crucial for designing effective interventions that address both disease burden and functional capacity (7, 10). To capture these multidimensional health states effectively, the EQ-5D-5L has emerged as a widely used standardized instrument that measures health by assessing five key dimensions: mobility (walking), self-care, usual activities, pain/discomfort, and anxiety/depression (11, 12).

Studies suggest that rural adults have significantly lower HRQoL compared to their urban counterparts, a disparity that is substantially pronounced by age, lower socioeconomic status, and the presence of chronic non-communicable diseases. These populations face unique challenges, including limited access to specialized healthcare facilities, geographical barriers, financial constraints, and reduced availability of specialized services (13). In the Indian context, this rural disadvantage is evident in data from the National Family Health Survey (NFHS-5, 2019–2021), which demonstrates a markedly higher prevalence of functional limitations among rural adults compared to urban populations (14).

Beyond structural factors, modifiable determinants such as tobacco use, nutritional status, and chronic diseases play a role in shaping HRQoL outcomes. The relationship between BMI and quality of life is often inverse (15, 16), while the presence of chronic diseases precipitates a cycle of reduced physical activity and further health decline (17). Despite this high burden of multimorbidity and functional outcomes in rural India, systematic documentation of HRQoL determinants remains limited. The existing literature primarily focuses on specific clinical cohorts (e.g., diabetes, hypertension, respiratory diseases) rather than general community-based populations. This knowledge gap limits the evidence base for designing population-level interventions and effectively prioritizing public health resources.

A comprehensive understanding of HRQoL provides the necessary evidence base for public health strategies aimed at enhancing quality of life in rural areas. The primary objective of this study was to assess health-related quality of life using the EQ-5D-5L index and determine its associated determinants among rural adults in western Maharashtra. By analyzing these factors in a large rural population, the research seeks to generate robust evidence for scalable interventions that address the specific health vulnerabilities of rural populations in similar low-resource contexts.

## Methods

### Study design

This was a cross-sectional study conducted in the rural areas of Pune district, Western Maharashtra, India.

### Study setting

The study was conducted in all 35 villages under a randomly selected *Male* Primary Health Centre (PHC) in Mulshi block, Pune district, Western Maharashtra, India. The PHC was selected by simple random sampling the four PHCs using a computer-generated random number.

**Figure 1:**
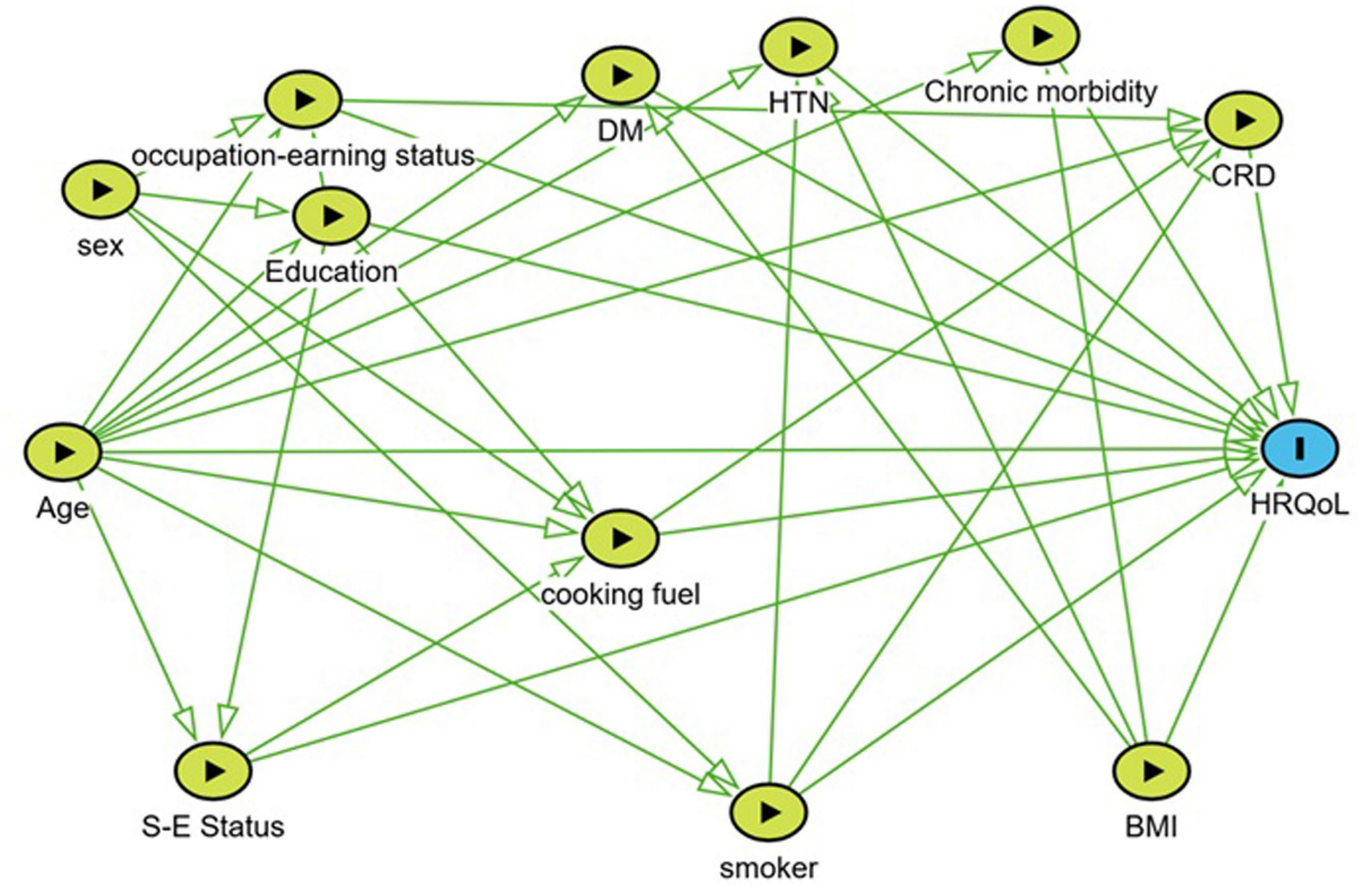
The directed acyclic graph (DAG) representing various predictors of the health-related quality of life (HRQoL). DM, diabetes mellitus; HTN, hypertension; CRD, chronic respiratory disease; HRQoL, health related quality of life; BMI, body mass index; S-E status, socioeconomic status.

Mulshi block is a semi-urban agricultural region with a mixed population of approximately 1,71,006 located in hilly area of Pune district. The region is characterized by agriculture (45%) as a primary occupation with rice as a main crop, small-scale manufacturing (30%), and the service sector (25%); there are four PHCs in the Mulshi block named *Male, Maan, Mutha*, and *Ambavane*, and one rural hospital (RH) at *Paud*. Except for *Ambavane*, which is a tribal PHC, the remaining PHCs cater to approximately 30–35 villages. These PHCs provide basic preventive, promotive, and curative services, including immunization, maternal health, family planning, and management of chronic diseases, such as diabetes and hypertension (18).

### Study participants

The study employed a census enumeration approach.

*Inclusion criteria*: Permanent residents of all the 35 villages aged 30 years and above who had lived in the village for at least 6 months and provided written informed consent.

*Exclusion criteria*: Individuals who could not be contacted on two consecutive visits (representing refusal, temporary migration, or unavailability).

The study population consisted of all enumerated eligible individuals from the selected PHC catchment area. The total number of eligible residents was 12,148. All participants meeting the inclusion criteria were invited to participate.

### Study duration

Data collection was conducted from April 1, 2024, to December 31, 2024.

*Data sources/measurements*: Data collection was conducted by 35 trained Accredited Social Health Activists (ASHA) workers, who are government-nominated voluntary health workers. The ASHAs received 2 days of training, including supervised practice sessions with detailed feedback, until they demonstrated 100% accuracy.

*ASHA training included*: (1) Informed consent procedures, questionnaire administration, and interview techniques. (2) Height and weight measurements were taken using a standardized stadiometer and digital weighing machine, respectively. (3) Calibration of weighing scales; (4) Peak expiratory flow rate (PEFR) measurement using a portable peak flow meter.

ASHAs visited all the permanent households and enrolled participants aged 30 years or above. They interviewed the eligible participants using a validated and pretested questionnaire.

## Variables studied

### Sociodemographic data

Age (completed years), gender, education (years of schooling, categorized as illiterate, 1–4 years, 5–10 years, and more than 10 years), and occupational status was categorized as (earning/non-earning).

Socioeconomic status was assessed using the color classification of ration cards, a government-recognized proxy in India directly linked to formal poverty line designations and access to welfare schemes. Classification included: yellow ration card (annual family income up to ₹15,000, below poverty line), orange ration card (annual income between ₹15,000–₹100,000), and white ration card (annual income above ₹100,000) (19).

### Anthropometry

The height and weight of the participants were taken in a standard manner, and body mass index (BMI) was assessed using the formula height in meters squared (kg/m^2^); the authors used World Health Organization (WHO) BMI categories to classify various categories of nutritional status (20).

Information regarding chronic disease was collected *via* self-report. Participants were presented with the question, “Have you received a physician diagnosis of any of the following chronic diseases?” and were instructed to select all applicable diseases from a provided list, allowing for multiple responses.

Participants’ perceptions of household ventilation were evaluated using their responses to a categorical question: “Do you consider the ventilation in your house to be adequate or inadequate?” Responses were classified into two categories: adequate or inadequate ventilation.

### Behavioral factors

The practices related to tobacco use were assessed through self-report. Participants were asked, “Do you smoke or consume smokeless tobacco products such as *gutka* or *khaini*?” Responses were categorized as never, past/occasional, or daily use. If the response was past/occasional, or daily use, further questions were asked about the type of tobacco, whether smoked or smokeless.

The type of fuel used for cooking was assessed through the question: “What kind of fuel do you use for cooking most of the time?” The response was classified as liquefied petroleum gas (LPG), or “*chullah*” (using burning of biomass); it was further classified as clean and unclean fuel, respectively.

### Health-related quality of life

The EQ-5D-5L, a validated *Marathi* translated version, was used to assess health-related quality of life. Permission to use this instrument was obtained from the EuroQol Research Foundation (11). The EQ-5D-5L assesses five dimensions: walking/mobility, self-care, usual activities, pain/discomfort, and anxiety/depression. Each dimension has five response levels, in ascending order of severity, ranging from “no problem” (level 1) to “extreme problems” (level 5). For analytical purposes, responses were dichotomized into “no problem” (level 1) and “any problem” (levels 2–5). This approach was chosen for its clinical meaningfulness: any reported problem, regardless of severity, indicates a functional limitation warranting health service attention. It aligns with EuroQol standard practice and published EQ-5D-5L guidance (11), providing adequate statistical power for subgroup analyses and facilitating comparison with other published studies. Utility scores were calculated using the Indian value set derived from the DEVINE (Developing an EQ-5D Value Set for India using an Extended design, ensuring culturally appropriate weighting of EQ-5D-5L dimensions for the Indian population) (21).

### Patient and public involvement

Prior to the study’s commencement, the cooperation of the village heads (Sarpanch) was obtained. The ASHA workers belonged to the same village community, making it easy to seek cooperation from the participants. Written informed consent was obtained from all participants before data collection.

### Bias and quality assurance

The interviews were conducted privately by trained ASHA workers who were familiar with local customs. We taught them to elicit correct information with emphasis on confidentiality. The familiarity between ASHAs and community members did not increase social desirability bias for sensitive variables, such as tobacco use, as the ASHA workers were fully aware of the habits of the individuals belonging to their allotted households. This was further mitigated by ensuring confidentiality. Quality assurance was ensured by the use of validated pretested study tool; and detailed quality checks throughout the data collection period through random visits of the field supervisor as well as investigators and cross-checking and verification of 5% of interviews; verification of data entry against source documents, range checks for all continuous variables, cross-validation of related variables, and supervisor review of a subset of completed questionnaires.

### Study size

Sample size was calculated using the formula for estimating a single proportion: *n* = *Z*^2^*α*/2 × *p*(1-*p*)/*d*^2^, where Z^2^*α*/2 = 3.84 (for 95% confidence interval, two-tailed alpha of 0.05), we used a precision of 5% of the expected prevalence of 14% from the DEVINE Study (21). Using a 95% confidence level and accounting for a 15% attrition rate, the required sample size was 11,106. We anticipated 15% attrition based on the author’s prior experience of working in the same community, temporary migration during the agricultural lean season, individuals being unavailable after two consecutive attempts, and participants who declined to participate.

However, this study employed a census enumeration approach, i.e., all 12,148 eligible permanent residents of the selected *Male* PHC catchment meeting the inclusion criteria were enumerated and invited to participate. This comprehensive census approach offered several methodological advantages, including the elimination of sampling variability and selection bias inherent in probability sampling. This approach, although deviating from the initial sample size calculation, enhances the rigor of the study.

### Statistical methods

Descriptive statistics, the chi-square test, *t*-test, and ANOVA test analyses were conducted using SPSS software (version 29.0; IBM Corp., Armonk, NY, USA). The prevalence ratio and adjusted prevalence ratio were determined using a modified Poisson regression model in Stata software (version 15.1; Stata Corp, College Station, TX, USA). Quantitative (continuous) variables, including the EQ-5D-5L index score (utility score), are summarized as the mean ± standard deviation (SD). Qualitative (categorical) variables are presented as frequencies and percentages. Differences in mean EQ-5D-5L index scores across sociodemographic variables, BMI, behavioral factors, and chronic diseases were assessed using independent-samples *t*-tests or one-way analysis of variance (ANOVA), as appropriate.

In addition to *p*-values, effect sizes were reported to quantify the magnitude of between-group differences. Initial bivariate associations between categorical variables and reporting “any problem” in any of the EQ-5D-5L dimensions (mobility/walking, self-care, usual activities, pain/discomfort, and anxiety/depression) were examined using chi-square tests. Crude (univariable) prevalence ratios (PRs) with 95% confidence intervals (CIs) were first estimated, followed by multivariable modified Poisson regression models with robust variance to obtain adjusted PRs and 95% CIs for the associations between socio-demographic and other factors and having “any problem” in each EQ-5D-5L dimension. The dichotomization of any problems versus no problem aligns with the EuroQoL guidance and published practice for EQ-5D-5L analysis (11). This binary classification addresses any deviation from full health.

Prevalence ratios were used instead of odds ratios to provide more intuitive measures of association for common outcomes. The modified Poisson models assume a correctly specified log-link function, independence of observations, and no severe multicollinearity among predictors. Robust (sandwich) standard errors were applied to account for potential misspecification of the variance structure when modeling binary outcomes. Multicollinearity was assessed and ruled out, as all Variance inflation factors (VIFs) were found to be below 2.5. All statistical tests were two-sided, and a *p*-value < 0.001 was considered statistically significant. The Indian value set determined the utility scores (21). Missing data was minimal, i.e., less than 1% and was handled considering complete case analysis. In our study, we intentionally used a significance level of 0.001. We chose this more conservative threshold due to the large sample size (*n* = 11,348). With such a large dataset, even minimal differences can become statistically significant at the 0.05 or 0.01 levels, which increases the risk of detecting statistically substantial but practically negligible effects. By setting the alpha level at 0.001, we aimed to reduce the likelihood of Type I errors (false positives) and ensure that only associations with greater statistical significance were considered statistically significant.

## Results

A total of 11,348 adults aged 30 years or older from 35 villages under *Male* PHC participated in this study, with a response rate of 93.4%. The mean (SD) EQ-5D-5L index score was 0.94 ± 0.17. Out of the total participants, 4,083 (36.8%) reported impairment in at least one dimension.

### Clinical and demographic characteristics of the study participants

Out of the total, 8,046 (71.90%) participants were in the 30–60 years age group, with males comprising 5,302 (46.7%) of the participants. The socioeconomic profile revealed that 457 (4.0%) participants belonged to the category below the poverty line (i.e., those with a yellow ration card). Over one-fourth, i.e., 3,224 (28.4%) of participants were illiterate. Out of the total, 5,036 (44.5%) were non-earning. A total of 2,233 (19.7%) reported using unclean fuel for cooking, and 3,908 (34.4%) used tobacco in some form; the majority, i.e., 3,770 (96.5%), used smokeless tobacco. Most participants, i.e., 6,139 (54.2%), had a normal nutritional status with a BMI of 18.5–24.99 kg/m^2^; 1,560 (13.7%) had at least one chronic morbidity. Hypertension was the most reported morbidity among 1,085 (9.6%) participants, followed by diabetes mellitus in 659 (5.8%) (Table 1).

**Table 1:** The mean EQ-5D-5L index score and characteristics of the rural adult participants aged 30 years and above (*n* = 11,348).

Characteristics	Category	EQ-5D-5L index			*p*-value	Effect size: (Cohen’s d/eta-squared)
		Frequency (%)	Mean	SD		
	Score	11,348	0.94	0.17		
EQ-5D-5L index	<0	90 (0.8)				
	0–0.99	4,083 (36.0)				
	1	7,175 (63.2)				
Age (years)	30–60	80,469 (70.9)	0.96	0.1	<0.001	0.16
	>60	3,302 (29.1)	0.87	0.25		
Ration card color	Yellow	457 (4.0)	0.95	0.12	0.12^c^	0.166
	Other	10,891 (96.0)	0.94	0.17		
Gender	Male	5,302 (46.7)	0.94	0.16	<0.001	0.166
	Female	6,046 (29.1)	0.93	0.17		
Education (years of schooling)	0 (Illiterate)	3,224 (28.4)	0.89	0.22	<0.001	0.040
	1–4	2,325 (20.5)	0.92	0.17		
	5–10	4,105 (36.2)	0.96	0.13		
	>10	1,694 (14.9)	0.98	0.09		
Occupation^a^	Earning	6,281 (55.5)	0.95	0.14	<0.001	0.165
	Non-earning	5,036 (44.5)	0.92	0.19		
Type of fuel	Unclean	2,233 (19.7)	0.92	0.15	<0.001	0.166
	Clean	9,115 (80.3)	0.94	0.17		
Tobacco use	Yes	3,908 (34.4)	0.91	0.19	<0.001	0.165
	No	7,440 (65.6)	0.95	0.15		
Type of tobacco	Smoking	138 (3.5)	0.87	0.28	0.02^c^	0.190
	Smokeless	3,770 (96.5)	0.91	0.19		
BMI^a^^,^^b^	<18.5	1,070 (9.4)	0.91	0.2	< 0.001	0.003
	18.5–24.99	6,139 (54.2)	0.94	0.15		
	25.0–29.99	3,211 (28.4)	0.95	0.14		
	≥30	903 (8.0)	0.94	0.16		
Chronic morbidity	Yes	1,560 (13.7)	0.86	0.28	< 0.001	0.163
	No	9,788 (86.3)	0.95	0.13		
Chronic respiratory disease	Yes	115 (1.0)	0.77	0.38	<0.001	0.165
	No	11,233 (99.0)	0.94	0.16		
Diabetes mellitus	Yes	659 (5.8)	0.89	0.24	<0.001	0.166
	No	10,689 (94.2)	0.94	0.16		
Hypertension	Yes	1,085 (9.6)	0.87	0.26	<0.001	0.164
	No	10,263 (90.4)	0.94	0.15		

a Total less than 11,348.

b Body mass index.

c No statistically significant difference, written as actual *p* value.

### EQ-5D-5L dimensions

Table 2 reveals that the most frequently affected dimension was pain/ discomfort for 3,212 (28.3%) participants, followed by anxiety/depression for 2,487 (21.9%).

**Table 2:** EQ-5D-5L frequencies and proportions reported by five dimensions and five dimensions among rural adult participants among rural adult participants aged 30 years and above (*n* = 11,348).

Problem (level code)	EQ-5D-5L dimensions				
	Walking/mobility no. (%)	Self-care no. (%)	Usual activities no. (%)	Pain/discomfort no. (%)	Anxiety/depression no. (%)
No (1)	9,396 (82.80)	10,664 (93.97)	9,691 (85.40)	8,136 (71.70)	8,861 (78.08)
Slight (2)	1,484 (13.08)	541 (4.77)	1,324 (11.67)	2,374 (20.92)	1950 (17.18)
Moderate (3)	237 (2.09)	70 (0.62)	199 (1.75)	615 (5.42)	404 (3.56)
Severe (4)	193 (1.70)	46 (0.41)	83 (0.73)	149 (1.31)	80 (0.70)
Extreme (5)	38 (0.33)	27 (0.24)	51 (0.45)	74 (0.65)	53 (0.47)

### Predictors of EQ-5D-5L impairment

The age above 60 years (adjusted PR, 1.42; 95% CI, 1.34–1.52), the use of unclean cooking fuel (adjusted PR, 1.18; 95% CI, 1.11–1.25), illiteracy (adjusted PR, 2.49; 95% CI, 1.78–3.48) as well as schooling up to 10 years, non-earning status 2.47 (95% CI, 1.67–3.67), presence of any chronic morbidity (PR 1.77, 95% CI 1.68–1.86) were significantly associated with impairment in EQ-5D-5L in multivariate models. The association of sex, BMI, chronic respiratory disease, diabetes mellitus, and hypertension was not sustained in the multivariate model (Table 3; Figure 1).

**Table 3:** The univariate and multivariate analyses of the prevalence ratio assessing the predictors of the EQ-5D-5L index score among rural adult participants aged 30 years and above.

Predictors	Categories	EQ-5D-5L		Total	Chi-square value	*p*-value	PR (95% CI)	Adjusted PR (95% CI)
		Some problem (level 2–4)	No problem (level 1)					
Age (years)	>60	1989	1,313	3,302	1102.7	<0.001	**2.21 (2.12–2.32)**	**1.42 (1.34–1.52)**
	30–60	2,184	5,862	8,046			1	1
Sex	Female	2,435	3,611	6,046	68.24	<0.001	**1.23 (1.17–1.29)**	1.04 (0.95–1.14)
	Male	1738	3,564	5,302			1	1
Ration card	Yellow	169	288	457	0.01	0.93^c^	1.01 (0.89–1.14)	0.75 (0.69–0.88)
	Other	4,004	6,887	10,891			1	1
Cooking fuel	Unclean	1,172	1,061	2,233	295.19	<0.001	**1.59 (1.52–1.67)**	**1.18 (1.11–1.25)**
	Clean	3,001	6,114	9,115			1	1
Education (years of schooling)	0 (Illiterate)	1793	1,431	3,224	1250.76	<0.001	**4.71 (4.12–5.38)**	**2.49 (1.78–3.48)**
	1–4	1,100	1,225	2,325			**4.01 (3.49–4.60)**	**2.51 (1.80–3.50)**
	5–10	1,080	3,025	4,105			**2.23 (1.94–2.56)**	**1.88 (1.34–2.63)**
	>10	200	1,494	1,694			1	1
Occupation^a^	Non-earning	2092	2,944	5,036	85.9	<0.001	**1.26 (1.20–1.32)**	**1.12 (1.03–1.222)**
	Earning	2078	4,203	6,281			1	1
Type of tobacco	Smoking	68	70	138	0.72	0.39	0.93 (0.78–1.11)	0.91 (0.76–1.08)
	Smokeless	1996	1774	3,770			1	1
BMI^a^^,^^b^	<18.5	489	581	1,070	73.92	<0.001	**1.21 (1.12–1.30)**	1.01 (0.94–1.10)
	25.0–29.99	1,026	2,185	3,211			0.84 (0.79–0.89)	0.94 (0.87–1.02)
	≥30	312	591	903			0.91 (0.82–1.00)	1.01 (0.89–1.15)
	18.5–24.99	2,325	3,814	6,139			1	1
Any chronic morbidity	Yes	918	642	1,560	379	<0.001	**1.77 (1.68–1.86)**	**1.37 (1.23–1.51)**
	No	3,255	6,533	9,788			1	1
Chronic respiratory disease	Yes	81	34	115	56.62	<0.001	**1.93 (1.71–2.18)**	1.03 (0.85–1.29)
	No	4,092	7,141	11,233			1	1
Diabetes mellitus	Yes	334	325	659	58.22	<0.001	**1.41 (1.30–1.53)**	0.94 (0.84–1.06)
	No	3,839	6,850	10,689			1	1
Hypertension	Yes	604	481	1,085	184.22	<0.001	**1.60 (1.51–1.70)**	0.84 (0.76–0.94)
	No	3,569	6,694	10,263			1	1

a Total less than 11,348.

b Body mass index.

c No statistically significant difference, written as actual *p* value.Bold indicates significant impairment.

### Determinants of impairment across EQ-5D-5L dimensions

The age group of 30–60 years demonstrated a significant association with impairment across all five dimensions, with the highest impairment reported for walking (adjusted PR, 2.09; 95% CI, 1.84–2.38). Unclean fuel use was associated with impairment in usual activities (adjusted PR, 1.43; 95% CI, 1.27–1.62) and the pain/discomfort dimension (adjusted PR, 1.27; 95% CI, 1.18–1.37). Non-earning status was associated with impairments in usual activities and pain/ discomfort with APR of 1.47 (95% CI, 1.22–1.76) and 1.22 (95% CI, 1.09–1.36), respectively. Presence of at least one chronic morbidity was associated with impairment in walking (adjusted PR 1.56, 95% CI 1.29–1.89), self-care (adjusted PR 1.72, 95% CI 1.06–2.78), usual activities (adjusted PR 1.81, 95% CI 1.46–2.26), and pain/discomfort dimension (Adjusted PR 1.42, 95% CI 1.23–1.63) (Table 4).

**Table 4:** Multivariate analyses comparing the predictors with various dimensions of EQ-5D-5L among rural adult participants aged 30 years and above.

Predictors	EQ-5D-5L dimensions				
	Walking	Self-care	Usual activities	Pain/discomfort	Anxiety/depression
	Adjusted prevalence ratio (95% CI)				
Age >60 years	**2.09 (1.84–2.38)**	**1.76 (1.34–2.33)**	**1.96 (1.70–2.67)**	**1.59 (1.46–1.73)**	**1.47 (1.33–1.63)**
Female	1.13 (0.95–1.35)	0.66 (0.44–1.00)	0.92 (0.76–1.11)	1.05 (0.93–1.18)	1.14 (0.98–1.31)
Illiterate	**1.80 (1.10–2.90)**	1.15 (0.50–2.70)	**1.77 (1.01–3.09)**	**2.56 (1.68–3.92)**	**2.21 (1.43–3.42)**
Non-earning	**1.40 (1.91–1.64)**	**2.47 (1.67–3.67)**	**1.47 (1.22–1.76)**	**1.22 (1.09–1.36)**	1.02 (0.89–1.17)
Smokers^b^	**1.38 (1.06–1.81)**	1.27 (0.69–2.31)	1.08 (0.48–1.78)	0.94 (0.76–1.16)	0.74 (0.54–1.01)
Unclean cooking fuel	1.02 (0.91–1.15)	0.99 (0.75–1.30)	**1.43 (1.27–1.62)**	**1.27 (1.18–1.37)**	0.96 (0.86–1.07)
BMI^a^ > 30	1.20 (0.98–1.50)	0.96 (0.58–1.59)	0.73 (0.53–1.02)	1.01 (0.85–1.20)	0.83 (0.66–1.05)
Chronic morbidity	**1.56 (1.29–1.89)**	**1.72 (1.06–2.78)**	**1.81 (1.46–2.26)**	**1.42 (1.23–1.63)**	1.18 (0.97–1.44)
Chronic respiratory disease	**1.40 (1.02–1.91)**	**2.13 (1.14–3.99)**	1.28 (0.92–1.79)	1.05 (0.81–1.35)	1.13 (0.79–1.60)
Diabetes mellitus	0.93 (0.75–1.33)	0.88 (0.56–1.39)	0.93 (0.75–1.16)	0.92 (0.79–1.07)	1.08 (0.89–1.31)
Hypertension	0.89 (0.74–1.07)	1.08 (0.68–1.72)	0.94 (0.76–1.17)	0.89 (0.77–1.02)	0.88 (0.72–1.06)

a Body mass index.

b Dimension-wise analysis was undertaken due to its public health importance, despite being non-significant on univariate/multivariate analysis.Bold indicates significant impairment.

## Discussion

Our large cross-sectional census study of 11,348 rural adults assessed health-related quality of life using the validated EQ-5D-5L instrument and identified key predictors that were associated with functional limitations across multiple dimensions. Our findings reveal an overall high mean index score of 0.94 (SD 0.17).

An apparent contradiction was found between the overall high mean index score (0.94) and the high prevalence of dimension-specific impairment. This can be explained by the fact that the EQ-5D-5L index score is a weighted composite utility measure that may not fully reflect the clinically significant impairment in individual domains. The pain/discomfort and anxiety/depression domains show substantial functional limitations. This highlights the importance of domain-specific interventions rather than relying solely on composite utility measures, such as the EQ-5D-5L, for public health planning.

The dimension-specific analysis revealed that the pain/discomfort dimension was most frequently affected in 3,212 (28.3%) individuals. This pattern aligns with findings from other studies (21–24). The high prevalence of pain-related functional impairment suggests musculoskeletal disorders that affect the rural agricultural population, substantially impairing work capacity and routine activities (23, 24). This highlights the public health prioritizing for pain management and mental health support as key components of comprehensive primary healthcare in rural populations. The next affected dimension was anxiety/depression for 2,487 (21.9%) participants. This mental health burden in rural areas reflects not only clinical psychiatric conditions but also distress related to poverty, agricultural uncertainties due to fluctuating income, crop failures, economic crisis, limited healthcare access, and other social determinants of mental health (25–27).

Age was strongly associated with HRQoL impairment, with individuals aged above 60 years having twice the prevalence of impairment compared to those under 60 years. These findings are consistent with those of other studies, which have reported that a progressive loss of muscle mass and a decrease in physical activity capacity are common in older populations (28, 29). Studies conducted elsewhere have reported that the walking/mobility dimension has the highest impairment in older adults (30, 31). These findings underscore the need for geriatric health programs, including the provision of assistive devices and physical rehabilitation services in rural primary healthcare settings.

Education emerged as a powerful determinant of HRQoL. Illiterates and those with less than 10 years of schooling were found to have significantly higher HRQoL impairment compared to those with more than 10 years of education, similar to a study among Brazilian older adults, which reported poor quality of life rating among those with lower education levels, while the urban–rural gap in HRQoL narrowed among older adults with higher education levels in a study in China (13, 31). Education opens up employment opportunities, increases earning potential, and improves access to information and healthcare services (32). These findings support education-focused interventions, including adult literacy programs and health literacy improvement, as critical public health strategies in rural areas.

A strong association has been found between the use of unclean fuel and the overall quality of life in the current study. The impairment in usual activity and pain/discomfort dimension of EQ-5D-5L may reflect chronic exposure to indoor air pollution from biomass fuel combustion, similar to other studies (33, 34). Exposure to household air pollution from unclean fuel causes respiratory inflammatory effects, reduced pulmonary function, and symptoms of dyspnoea and cough, which directly impact usual activities and indirectly affect psychological well-being (34, 35). The fact that nearly one in five adults (19.7%) in the current rural cohort still uses unclean fuel for cooking represents a public health concern, thereby representing a significant number of rural populations at risk for impairment in HRQoL. Although LPG subsidies and other schemes are existing public health interventions promoting clean cooking fuels, awareness and culturally appropriate education are needed to maximize utilization and sustain clean fuel adoption in rural communities (36, 37).

In our study, participants with at least one chronic morbidity had a significant association with impairment in all dimensions of HRQoL. Access to healthcare in rural areas is limited due to long distances, fewer facilities, and limited financial resources (32, 38). Consequently, many chronic conditions may remain undiagnosed or inadequately treated, thereby reducing the overall quality of life (39–41).

The “presence of any chronic comorbidity” category (*n* = 1,560) represents individuals with at least one chronic condition, which is smaller than the sum of individual conditions because of the presence of multiple comorbidities. The separate dichotomous classifications enable the examination of both the overall burden of chronic disease and the specific, independent effects of individual conditions after adjusting for multiple variables. The “non-diseased” groups are not disease-free, but they represent individuals who either do not have that particular condition or have alternative chronic disease profiles, which explains the discrepancy between individual condition counts and total comorbidity counts. This approach enables assessment of whether certain conditions have unique associations with health-related quality of life domains beyond the general effect of having any chronic disease.

In multivariate analysis, the presence of chronic respiratory disease (CRD) showed weaker associations with the EQ-5D-5L index score. This is likely due to the under diagnosis of these chronic conditions in rural settings, thereby impairing the quality of life (42, 43). In contrast, the studies done among rural adults reported significantly poor quality of life and some form of functional disability among patients with COPD and asthma (44–46). The walking and self-care dimension showed the most substantial impairment for the individuals with CRD. Chronic respiratory diseases, i.e., COPD and asthma, have recently been added to the government program on non-communicable diseases; however, their implementation is yet to occur (47).

Early asymptomatic hypertension and diabetes may not be recognized as a disease by affected individuals. Many studies have established that diabetes significantly impairs quality of life (39, 48); these findings are consistent with our findings that rural diabetic adults have lower health-related quality of life scores than those without diabetes. Similarly, other studies have reported that hypertensive participants had significantly lower EQ-5D-5L indexes than non-hypertensive participants (49, 50). However, a strong association did not emerge from our research. Since diabetes and hypertension are managed free of cost under the National Non-communicable Diseases Program through ASHA workers, individuals with well-controlled chronic conditions treated with pharmacotherapy may not experience functional HRQoL impairment, thereby attenuating observed associations.

In the current study, the color of the ration card was used as an indicator of income. This approach was chosen because it involves objective government documentation that minimizes social desirability bias, thereby reflecting validated poverty classifications. All welfare programs are linked to the color of the ration card. Contrary to expectations, the current study found that income was not significantly associated with HRQoL, a finding consistent with a study conducted in China (13). This may be because only 4.0% of the population falls below the poverty line. The availability of Government Welfare schemes, such as Ayushman Bharat, pension schemes, and food security, which buffer low-income households, and non-linear income-HRQoL relationships may contribute to this. Education and occupation, analyzed separately, were significantly associated with HRQoL, suggesting that socioeconomic determinants of HRQoL may involve multiple interconnected pathways rather than income alone. At the same time, studies elsewhere have reported that better financial capacity improves HRQoL (30, 49). Thus, future studies employing continuous income measurement, stratified analysis by welfare scheme beneficiary status, and exploration of income-health interactions are warranted.

In adjusted analysis, gender was not a significant predictor of HRQoL impairment in our study, suggesting a more equitable gender based HRQoL in this specific rural population in contrast to other studies (31, 39). This indicates that the gender may not be the primary driver of HRQoL variation in this setting, even after accounting for age and socioeconomic factors.

In our study, overweight rural adults had a higher risk of impairment than other categories, as supported by a study done elsewhere (40). Nutritional status, assessed via BMI, exhibited an interesting pattern, where overweight individuals (BMI 25.0–29.99 kg/m^2^) had higher mean EQ-5D-5L scores compared to individuals in other BMI categories. This may reflect the improved nutritional status in overweight individuals compared to underweight individuals, or individuals with chronic diseases, who may lose weight. Additionally, measuring BMI at a single time point does not capture longitudinal nutritional trends. The elevated impairment in underweight individuals likely reflects both nutritional deficiency and potential chronic wasting diseases. Conversely, obesity may be associated with a decline in HRQoL due to mobility limitations and comorbidities.

Only the type of tobacco use, i.e., smoking or smokeless, was analyzed in both univariate and multivariate analyses. There is a substantial reduction in health-related quality of life with tobacco use and diseases attributed to its use as reported in various studies (51). However, in the current study, it was not retained as a statistically significant independent predictor in multivariate models. The dimension-specific effects may be masked in the composite EQ-5D-5L index, as there is a possibility of underreporting of tobacco use, despite anonymity and privacy measures, driven by social norms and gender expectations in rural communities. Although we did not find a significant independent association in this study, tobacco control remains a general public health priority.

BMI, CRD, hypertension, and diabetes mellitus, which were significantly associated in the univariate model, lost significance after adjustment. This suggests that sociodemographic factors likely confounded their initial association. Once these factors were controlled, the independent effect of BMI and CRD, hypertension, and diabetes mellitus on HRQoL was attenuated. This highlights the importance of adjusting for socioeconomic confounders to avoid overestimating the impact of nutrition (assessed using BMI) and chronic diseases.

The study’s strength lies in its comprehensive census enumeration of the entire eligible population (11,348 participants), which eliminates sampling bias and provides population-level estimates suitable for health program planning. Additionally, the use of the validated EQ-5D-5L Marathi version with Indian value sets ensures precise, culturally appropriate, and standardized measurement of health-related quality of life. The instrument is more suitable for rural areas, as it has a lower respondent burden compared to other instruments (52, 53). The large study sample size makes our results more authentic and reliable. Systematic data collection by trained ASHAs with quality checks, minimal missing data (<1%), supporting analytical integrity. Integration of multiple dimensions of health and well-being (sociodemographic, behavioral, morbidity, and environmental) is done in this single study framework. The study provides insights directly translatable into public health and program prioritization to improve the quality of life of rural residents. The study was conducted within a single PHC catchment area in Western Maharashtra, caution should be exercised while generalizing the findings to the national level as variation in cultural practices, access to health services, and environmental exposures may limit its application to other rural areas across India. The results should be interpreted as indicative of similar low resource context rather than universal representative.

The study’s limitations include the cross-sectional design, which limits causal inference regarding temporal relationships.

The binary classification of EQ-5D-5L resulted in a loss of ordinal granularity; however, this was necessary, as there was sparse data in the severe to extreme categories, which would have made the ordinal regression estimates unreliable.

The presence of chronic disease was self-reported, which might have introduced misclassification bias due to the large number of undiagnosed morbidities, thereby lowering the HRQoL scores of the reference group. This misclassification would have likely biased the regression coefficient toward null, suggesting that the presence of chronic diseases might be significantly associated with HRQoL. Some sociodemographic variables (earning status) may represent confounders, mediators, or chronic disease consequences rather than independent causes. Associations between predictors and HRQoL should not be interpreted as causal. Additionally, some of the working male population may be underrepresented. Socioeconomic status measurement via ration card color represents a crude proxy that does not capture income variation above the poverty threshold.

The future studies should investigate the longitudinal trajectories of HRQoL in rural populations. Implementation studies are needed to assess how to integrate routine HRQoL monitoring into primary health care. In conclusion, this large-scale rural community study demonstrates that the strongest factors associated with HRQoL impairment are illiteracy, age greater than 60 years, the presence of chronic disease, and the use of unclean fuel. Thus, these are identifiable at-risk groups requiring targeted interventions. Several of these factors are amenable to public health action, including improving education and health literacy, implementing tobacco cessation programs, increasing the number of households that transition to clean cooking fuels, early detection and management of chronic diseases, and integrating HRQoL monitoring into routine primary healthcare.

## Data Availability

The raw data supporting the conclusions of this article will be made available by the authors, without undue reservation.
