# Correction: Predictors of health-related quality of life among rural adult residents of western Maharashtra, India

**DOI:** 10.3389/fpubh.2026.1894691

**Published:** 2026-07-01

**Authors:** Jayashree Gothankar, Sanjivani Patil, Medha Bargaje, Rupeshkumar Deshmukh, Ashwini Devane, Prakash Doke

**Affiliations:** 1 Department of Community Medicine, Bharati Vidyapeeth Deemed University Medical College, Pune, India; 2 Central Research and Publication Unit, Bharati Vidyapeeth Deemed University Medical College, Pune, India; 3 Department of Pulmonary Medicine, Bharati Vidyapeeth Deemed University Medical College, Pune, India

**Keywords:** BMI, chronic disease, EQ-5D-5L, health-related quality of life, predictors, rural adults, rural health

In the published article, there was an error in the main text citations. The sentence previously stated “There is a substantial reduction in health-related quality of life with tobacco use and diseases attributed to its use as reported in various studies (31,40,50,51).” The correct citation should be “There is a substantial reduction in health-related quality of life with tobacco use and diseases attributed to its use as reported in various studies (51).”

In the published article, there was an error in the main text citations. The sentence previously stated “Early asymptomatic hypertension and diabetes may not be recognized as a disease by affected individuals. Many studies have established that diabetes significantly impairs quality of life (27,39,48).” The correct citation should be “Early asymptomatic hypertension and diabetes may not be recognized as a disease by affected individuals. Many studies have established that diabetes significantly impairs quality of life (39,48).”

In the published article there was a mistake in the references. The reference for [15] was erroneously written as “Endo T, Lee XJ, Clemens SL. EQ-5D-5L population norms and quality-adjusted life expectancy by sociodemographic characteristics and modifiable risk factors for adults in Queensland, Australia. Value Health. (2024) 27:633–41. doi: 10.1016/j.jval.2023.12.014”. The correct reference should be “Endo T, Lee XJ, Clemens SL. EQ-5D-5L population norms and quality-adjusted life expectancy by sociodemographic characteristics and modifiable risk factors for adults in Queensland, Australia. *Value Health*. (2024) 27:633–41. doi: 10.1016/j.jval.2024.02.007”

In the published article there was a mistake in the references. The reference for [27] was erroneously written as “Sang S, Liao W, Kang N, Wu X, Hu Z, Liu X, et al. Health-related quality of life assessed by EQ-5D-5L and its determinants among rural adults: results from the Henan rural cohort study. Eur J Health Econ. (2024) 25:21–30. doi: 10.1007/s10198-023-01565-y” The correct reference should be “Kirkbride JB, Anglin DM, Colman I, Dykxhoorn J, Jones PB, Patalay P, et al. The social determinants of mental health and disorder: evidence, prevention and recommendations. *World Psychiatry*. (2024) 23:1. doi: 10.1002/wps.21160”

In the published article there was a mistake in the references. The reference for [35] was erroneously written as “Lim SS, Murray CJL, Gakidou E, et al. Association between biomass cooking fuels and prevalence of tuberculosis in India: a community-based cross-sectional study. PLoS Med. (2024) 12:e1004356. doi: 10.1371/journal.pmed.1004356” The correct reference should be “Mishra VK, Retherford RD, Smith KR. Biomass cooking fuels and prevalence of tuberculosis in India. *Int J Infect Dis*. (1999) 3:119–29. doi: 10.1016/s1201-9712(99)90032-2”

The original version of this article has been updated.

